# The Application of LC-MS in Pharmaceutical Analysis

**DOI:** 10.3390/molecules31152624

**Published:** 2026-07-28

**Authors:** Wing Lam, Wei Cai

**Affiliations:** 1 Department of Pharmacology, Yale University School of Medicine, New Haven, CT 06510, USA; 2 School of Pharmaceutical Sciences, Hunan University of Medicine, Huaihua 418000, China

## 1. Introduction

Liquid chromatography–mass spectrometry (LC–MS) has evolved from a specialized analytical tool into an indispensable cornerstone of pharmaceutical analysis over the past three decades. Continuous refinements in liquid chromatography—from conventional HPLC to UHPLC and UHPLC—coupled with dramatic advances in mass spectrometry, particularly high-resolution Orbitrap and quadrupole-Orbitrap platforms, have endowed analysts with unprecedented sensitivity, selectivity, and structural resolution. The maturation of triple quadrupole tandem MS (QqQ-MS) for targeted quantification, time-of-flight (Q-TOF) and quadrupole-time-of-flight (Qq-TOF) systems for untargeted profiling, and the development of novel ionization sources and chromatography modes, have collectively expanded the scope of LC–MS far beyond its original boundaries.

The Special Issue “The Application of LC–MS in Pharmaceutical Analysis” was launched to provide a focused platform for disseminating methodological innovations and charting the current state of the field across pharmaceutical science. We envisioned a collection spanning the full analytical pipeline—from method development and validation, through chemical characterization and impurity profiling, to pharmacokinetic analysis, metabolomics, and high-throughput drug discovery. The enthusiastic response from the global scientific community has produced a themed collection of 13 articles that collectively illustrate the breadth, depth, and vitality of contemporary LC–MS applications in pharmaceutical analysis.

## 2. Method Development and Validation

Rigorous method development and validation remain foundational requirements for regulatory compliance, therapeutic drug monitoring, and bioequivalence assessment. Four contributions in this Special Issue exemplify the current state of the art, with each addressing distinct analytical challenges and demonstrating the adaptability of LC–MS.

Song et al. [Contribution 1] developed a highly sensitive LC–MS/MS assay for the precise quantification of sitagliptin in human plasma, achieving a total runtime of under 2 min using isocratic elution on a Kinetex^®^ C18 column. The method required only 100 µL of plasma with MTBE liquid–liquid extraction and was comprehensively validated against both MFDS (Republic of Korea) and FDA criteria, demonstrating excellent linearity (5–1000 ng/mL, r^2^ > 0.998), accuracy, precision, and stability. This exemplifies the ongoing drive toward micro-scale, high-throughput bioanalytical methods for clinical and regulatory environments.

Karubothula et al. [Contribution 2] developed an automated LC–MS/MS method for the simultaneous detection of 69 pharmaceutical and personal care products (PPCPs) in wastewater, utilizing a Biomek i7 robotic workstation for 96-well plate SPE. The method achieved ±20% matrix effect tolerance for 63 of 69 targets, with recovery of 80–120% and %RSD ≤ 20%. Evaluated using the Green Analytical Procedure Index (GAPI) and Blue Applicability Grade Index (BAGI), the approach bridges pharmaceutical analysis with environmental public health surveillance.

El Orche et al. [Contribution 3] presented a fully ICH M10-compliant validation of an LC–MS/MS method for fluoxetine quantification in human plasma, with an applied pharmacokinetic study. This work is particularly timely given the increasing global enforcement of ICH M10 guidelines and the need for harmonized bioanalytical standards across regulatory jurisdictions.

Su et al. [Contribution 4] reported a combined in silico and in vivo pharmacokinetic evaluation of 84-B10, a novel 3-phenylglutaric acid derivative active against acute kidney injury (AKI) and chronic kidney disease (CKD). Integrating computational ADME prediction with empirical in vivo validation, this dual approach exemplifies a rational strategy in early drug discovery for prioritizing lead compounds for further development.

Collectively, these four studies underscore three contemporary themes: (i) miniaturization and micro-scale sample requirements for clinical applications; (ii) automation and high-throughput capacity enabled by robotic sample preparation; and (iii) the integration of green analytical chemistry principles and regulatory compliance standards into method development.

## 3. Chemical Characterization of Complex Matrices

Chemical characterization of natural products and botanical matrices demands both chromatographic resolution of highly similar components and sensitive, information-rich detection. Three contributions in this Special Issue demonstrate complementary LC–MS strategies for distinct characterization challenges.

Yang et al. [Contribution 5] presented a systematic UHPLC-Q-Exactive Orbitrap MS method for the identification of oligosaccharide constituents in *Polygonatum cyrtonema* Hua (PCH), a common medicinal and edible plant. Combining UHPLC-Q-Exactive Orbitrap MS with monosaccharide composition analysis, 44 oligosaccharides—including 27 fructo-oligosaccharides (FOS), 10 arabino-oligosaccharides (AOS), and seven others—were successfully identified, all reported for the first time in *P. cyrtonema* Hua. This work demonstrates the power of UHPLC-HRMS for deconvolution of complex carbohydrate mixtures beyond the reach of conventional UV detection.

Huo et al. [Contribution 6] established an efficient UHPLC-Q-Exactive Orbitrap MS method for the comprehensive chemical profiling of Erdun-Uril, a Mongolian traditional medicine compound comprising 29 herbs used for cerebrovascular ailments. This work addresses a fundamental challenge in traditional medicine research: systematically characterizing the chemical constituents of polyherbal preparations whose pharmacological effects arise from synergistic interactions among multiple bioactive components.

Zhang et al. [Contribution 7] addressed TCM authentication and adulterant detection, developing an LC–MS method combined with chemometrics for Rubiae Radix et Rhizoma (RRR) and its common adulterants Rubia schumanniana (RSP) and Rubia magna (RMP). The authors introduced an innovative “ion identity” concept: ion intersections and de-intersections across multiple batches were used to identify proprietary ions characteristic of each species, yielding a recognition index (RI) that enabled objective authentication. The method detected 3% adulteration levels and identified three market samples as adulterants when combined with PCA and OPLS-DA—a conceptually elegant and computationally straightforward approach adaptable to other herbal medicines.

## 4. Metabolomics

Metabolomics—the comprehensive profiling of small-molecule metabolites in biological systems—has become one of the most impactful applications of LC–MS. Four contributions in this Special Issue push the boundaries of metabolomic profiling from complementary angles.

Xie et al. [Contribution 8] reported a sophisticated mid-level data fusion strategy combining UPLC-Q/Orbitrap MS (non-volatile metabolites) with headspace SPME GC–MS (volatile metabolites) to differentiate green Forsythiae Fructus (GFF) from ripe Forsythiae Fructus (RFF). The OPLS-DA model built on fused mid-level data achieved superior predictive performance (R^2^Y = 0.986, Q^2^ = 0.974) compared to single-technique models. By applying VIP > 1 and *p* < 0.05 criteria to fused data, differential compounds were narrowed to 30—effectively reducing noise compared to the 61 initially identified by individual techniques. This exemplifies best-practice botanical metabolomics where both volatile and non-volatile constituents contribute to pharmacological activity.

Xu et al. [Contribution 9] developed and validated targeted LC–MS/MS methods for the simultaneous quantification of 235 plasma metabolites spanning 17 compound classes in porcine plasma—one of the largest targeted metabolomics panels reported using a dual-chromatography (RPLC + HILIC) approach without derivatization. A simple “dilute-and-shoot” protocol, with samples injected at two dilution levels to accommodate the approximately nine-order-of-magnitude concentration range, represents a practical workflow suitable for large-scale biomedical studies. Cross-validation across multiple animal species demonstrates broad applicability to comparative metabolic studies in veterinary and translational research.

Li et al. [Contribution 10] combined untargeted and targeted metabolomics to reveal that the active peptide of Eupolyphaga sinensis Walker (APE) exerts anti-hyperlipidemic effects by modulating imbalanced amino acid metabolism. After characterizing APE composition and evaluating its therapeutic effects on lipid levels, inflammatory response, and oxidative stress in hyperlipidemic rats, metabolomics delineated the specific metabolic pathway alterations underlying the therapeutic mechanism. This exemplifies the power of integrative metabolomics for linking a biochemically characterized natural product to specific in vivo metabolic pathway modulations in a disease model.

Anh et al. [Contribution 11] provided a comprehensive review of recent advances in mass spectrometry-based targeted metabolomics and lipidomics, systematically evaluating sample collection protocols, quantification strategies, quality control frameworks, and data interpretation pipelines. The authors chart a convincing trajectory for how LC–MS-powered targeted metabolomics and lipidomics are becoming indispensable tools for disease biomarker discovery, patient stratification, and precision medicine.

## 5. Drug Metabolism and Impurity Profiling

Understanding the metabolic fate of drugs and natural products is essential for establishing their pharmacological relevance and regulatory compliance. Two contributions address this critical interface.

Cui et al. [Contribution 12] developed a UHPLC-HRMS strategy in both positive and negative ion modes to comprehensively identify genipin metabolites—the aglycone of geniposide from Gardenia jasminoides Ellis—in rat urine, blood, feces, and fecal fermentation. By combining precise mass measurement, MS/MS fragmentation pattern analysis, and comparison with reference standards, the authors systematically characterized genipin’s metabolic pathways, providing direct material basis evidence for its pharmacological effects and a methodological template for metabolite identification of other iridoid compounds.

Wroński et al. [Contribution 13] investigated miconazole metabolism using a dual approach coupling human liver microsome (HLM) incubation and electrochemical oxidation to UHPLC-HRMS, identifying seven previously unreported metabolites, including products of benzene ring hydroxylation and imidazole moiety oxidation. The comparison between biological (HLM) and electrochemical oxidation systems demonstrated how electrochemical simulation can serve as a valuable predictive complement to conventional in vitro metabolic stability assessments.

## 6. Conclusions

The 13 articles in this Special Issue collectively demonstrate that LC–MS—in its various configurations, from QqQ-MS for targeted quantification to high-resolution Q-Orbitrap and Q-TOF for untargeted profiling—remains at the heart of pharmaceutical analysis. The contributions span method development with regulatory compliance; systematic chemical characterization of complex polyherbal preparations; large-scale plasma and botanical metabolomics; pharmacokinetic profiling of natural products and drug candidates; and environmental pharmaceutical residue monitoring.

Several convergent insights emerge. First, the analytical power of LC–MS is multiplied when combined with complementary techniques—whether GC–MS for volatiles, electrochemical simulation for metabolism prediction, chemometrics for data integration, or robotic automation for scalability. Second, HRMS has become the dominant platform across all application domains, with high mass accuracy (<5 ppm) and full-scan sensitivity enabling both retrospective data mining and confident compound annotation. Third, the transition from descriptive compositional analysis to mechanistic biological inquiry—using LC–MS to elucidate how botanical preparations are metabolized in vivo and how metabolic profiles relate to therapeutic outcomes—represents one of the most consequential evolutions in the field. We express our sincere gratitude to all authors, reviewers, and the editorial staff of *Molecules* for their rigorous evaluation and professional support.

